# Clinical, microbiological, and immunological effects of systemic probiotics in periodontal treatment: study protocol for a randomized controlled trial

**DOI:** 10.1186/s13063-021-05246-0

**Published:** 2021-04-15

**Authors:** Belen Retamal-Valdes, Wim Teughels, Laryssa Macedo Oliveira, Rebeca Nascimento da Silva, Aretuza Fritoli, Patricia Gomes, Geisla Mary Silva Soares, Natalie Temporão, Camila Pinheiro Furquim, Poliana Mendes Duarte, Helio Doyle, Marcelo Faveri, Luciene Cristina Figueiredo, Magda Feres

**Affiliations:** 1 Department of Periodontology, Dental Research Division, Centro de Pós-Graduação e Pesquisa-CEPPE, Guarulhos University, Praça Tereza Cristina, 229 Centro, Guarulhos, SP 07023-070 Brazil; 2 Department of Oral Health Sciences, Periodontology, Katholieke Universiteit Leuven (KULeuven), Leuven, Belgium; 3 Department of Stomatology, Federal University of Parana, Curitiba, Parana Brazil; 4 Department of Periodontology, College of Dentistry, University of Florida, Gainesville, FL USA

**Keywords:** Periodontitis, Probiotic, Metronidazole, Amoxicillin, Scaling and root planing, Treatment

## Abstract

**Background:**

The association of scaling and root planing (SRP) with systemic metronidazole (MTZ) plus amoxicillin (AMX) has shown to be an effective treatment protocol, particularly for periodontitis stages III and IV, generalized. More recently, probiotics have also been suggested as a promising adjunctive treatment for periodontal diseases due to their antimicrobial and anti-inflammatory properties. Therefore, the aim of this randomized clinical trial (RCT) is to evaluate the clinical, microbiological, and immunological effects of probiotics as adjuncts to SRP alone or with MTZ+AMX in the treatment of periodontitis.

**Methods:**

Subjects with periodontitis are being randomly assigned to receive (i) SRP alone, or with (ii) two probiotic lozenges/day for 90 days (Prob), (iii) MTZ (400 mg) and AMX (500 mg) thrice a day (TID) for 14 days (MTZ+AMX), or (iv) Prob and MTZ+AMX. Subjects are being monitored for up to 12 months post-treatment. Nine subgingival plaque samples per patient are being collected at baseline and at 3, 6, and 12 months post-therapy and analyzed by checkerboard DNA–DNA hybridization for 40 bacterial species. Peripheral blood and gingival crevicular fluid (GCF) of four randomly selected periodontal sites will be analyzed by means of a multiplex fluorescent bead-based immunoassay for 17 cyto/chemokines.

**Statistical analyses:**

The significance of differences in each group (over the course of the study) will be sought using repeated measures ANOVA or Friedman tests and among groups (at each time point) using either ANOVA/ANCOVA or Kruskal-Wallis tests, depending on normality of the data. The chi-square test will be used to compare differences in the frequency of subjects achieving the clinical endpoint for treatment (≤ 4 sites with PD ≥ 5 mm) at 1 year and of self-perceived adverse effects. A stepwise forward logistic regression analysis will be performed in order to investigate the impact of different predictor variables on the percentage of patients achieving the clinical endpoint for treatment. The Number Needed to Treat (NNT) with different treatment protocols will be also calculated. Statistical significance will be set at 5%.

**Trial registration:**

ClinicalTrials.gov NCT03733379. Registered on November 7, 2018.

**Supplementary Information:**

The online version contains supplementary material available at 10.1186/s13063-021-05246-0.

## Background

Scaling and root planing (SRP), the standard of care for treatment of periodontitis, leads to an overall improvement in clinical parameters, including reduction in probing depth (PD), percentage of sites with bleeding on probing (BOP), and gain in clinical attachment [1–3]. However, SRP does not lead to major and sustainable clinical improvements in all subjects and periodontal sites, especially in cases of severe periodontitis [1, 4–6]. This is probably because SRP alone is unable to produce a sufficiently deep change in the oral microbial ecology (rebiosis) in order to produce a new stable climax biofilm community compatible with periodontal health [1, 2, 6–9]. Therefore, adjunctive therapies, such as systemic antimicrobials [6, 10–19] and probiotics [20–24], have been proposed with the purpose of potentiating the effects of SRP.

Among all the systemic antibiotics tested to date in the treatment of periodontitis, the combination of metronidazole (MTZ) and amoxicillin (AMX) has been shown to offer the greatest clinical and microbiological benefits. There is consistent scientific evidence showing that the adjunctive use of MTZ+AMX improves the outcomes of SRP, including a greater reduction in the number of deep pockets after treatment and, consequently, in the need for surgical interventions [19]. A number of ecological benefits have been associated with the use of MTZ+AMX during the active phase of treatment. This adjunctive therapy is effective in reducing key periodontal pathogens, such as *Porphyromonas gingivalis* and *Aggregatibacter actinomycetemcomitans* [25–30] in periodontal pockets and on other oral surfaces, and to foster an increase in the proportions of beneficial microorganisms [6].

More recently, probiotics have also been suggested as promising adjunctive treatments for periodontitis [20–24, 31–35]. The effects of probiotics in periodontal treatment have been investigated in vitro and in vivo [36–38]. Subgingival recolonization by periodontopathogens [36] and the level of local inflammation [37] are delayed or prevented with the use of probiotics. Probiotics also had a favorable impact on bone density within periodontal pockets [39]. However, few randomized clinical trials (RCTs) have evaluated the effectiveness of systemic probiotics as adjuncts to SRP in periodontal treatment [40–48]. The results of these RCTs have indicated an overall improvement in clinical parameters with the adjunctive use of systemic probiotics in the treatment of periodontitis. However, to date, no robust RCT have evaluated the effects of systemic probiotics in periodontal treatment or have compared the outcomes of this treatment with those obtained with the adjunctive use of systemic antibiotics.

## Methods/design

### Aim, design, and setting of the study

The aim of this study is to evaluate the clinical, microbiological, and immunological effects of systemic probiotics as an adjunct to SRP alone or in combination with MTZ+AMX in the treatment of periodontitis. In order to address this aim, a double-blinded, four-armed, placebo-controlled, and bicentric RCT was designed. This study is being conducted at Guarulhos University (UNG; Guarulhos, SP, Brazil) and Federal University of Parana (UFPR; Curitiba, PR, Brazil). The study protocol was developed according to the Standard Protocol Items: Recommendations for Interventional Studies (SPIRIT) guidelines and using the SPIRIT checklist (Additional file 1) [49] and was registered in the ClinicalTrials.gov (NCT03733379) database.

### Ethical considerations

This trial is being conducted according to the principles of the Declaration of Helsinki for studies involving human subjects. The protocol was approved by the Institutional Review Board of UNG (Research Ethics Committee, CAAE: 74049717.7.1001.5506) and UFPR (Research Ethics Committee, CAAE: 74049717.7.3001.0102). All eligible volunteers are being informed about the nature, potential risks, and benefits of their participation in this study, and they sign a term of informed consent (Additional file 2).

### Subject population and inclusion/exclusion criteria

Systemically healthy volunteers with untreated generalized severe periodontitis (periodontitis stages III or IV, generalized [50];) are being selected from the Center for Clinical Trials of UNG and Periodontal Clinic of UFPR. Subjects are being selected according to the following inclusion criteria: presence of at least 15 teeth (excluding third molars and teeth indicated for extraction), at least 30% of the teeth with PD and CAL ≥ 4 mm, and a minimum of 6 teeth with at least one interproximal site each with PD and clinical attachment level (CAL) ≥ 5 mm. Exclusion criteria are as follows: pregnancy, breastfeeding, current smokers and former smokers—for at least 5 years [51], systemic diseases that could affect the progression of periodontitis (e.g., diabetes, immunological disorders, osteoporosis), SRP in the previous 12 months, antibiotic therapy in the previous 6 months, long-term intake of anti-inflammatory drugs, need for antibiotic pre-medication for dental treatment, use of orthodontic appliances, extensive prosthetic rehabilitation, and allergy to MTZ and/or AMX.

### Interventions

**Fig. 1:**
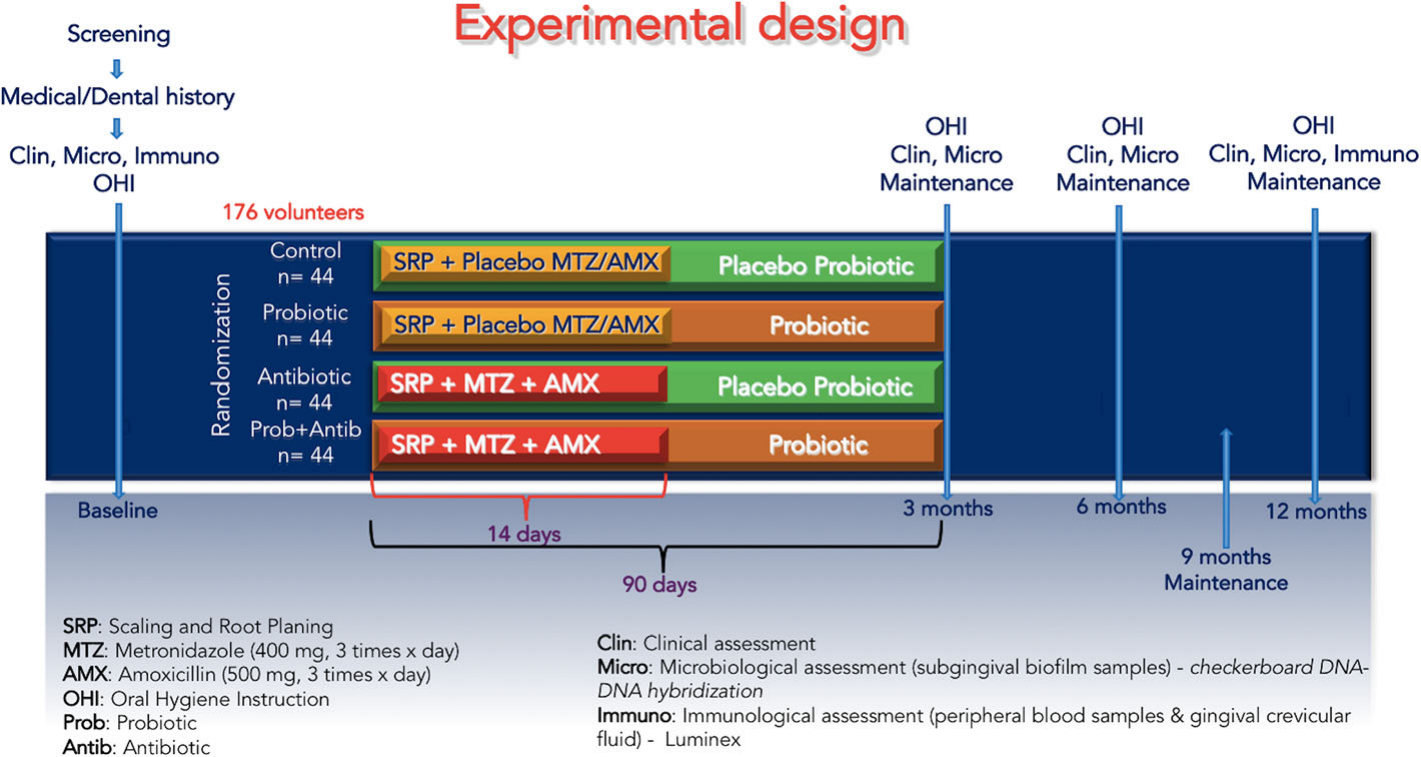
Experimental design of the study. AMX, amoxicillin (500 mg, 3 times × day); CLIN, clinical assessment; OHI, oral hygiene instruction; IMUNO, immunological assessment; MICRO, microbiological assessment; MTZ, metronidazole (400 mg, 3 times × day); Prob, probiotic; PMT, periodontal maintenance therapy; SRP, scaling and root planing

**Fig. 2:**
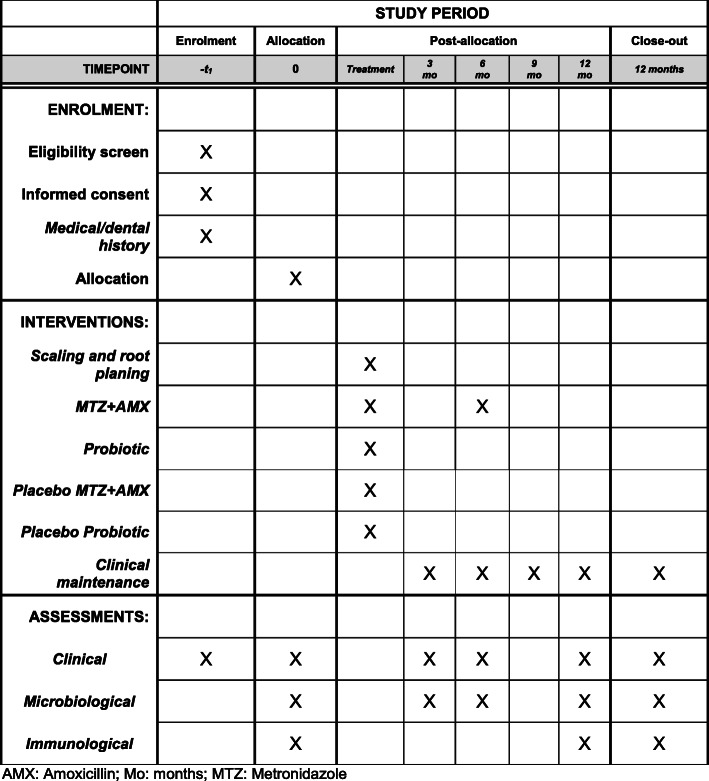
Schedule of volunteers’ enrolment, interventions, and assessments according to the SPIRIT statement. AMX, amoxicillin; Mo, months; MTZ, metronidazole

At baseline, all volunteers fulfill a structured questionnaire to collect information about demographic, oral, and general health data. Subsequently, a complete periodontal clinical assessment and subgingival biofilm sampling are performed. One week later, gingival crevicular fluid (GCF) and peripheral blood samples are collected. Patients receive oral hygiene instruction (OHI), including tooth brushing and interproximal dental hygiene (using dental floss and interdental brushes). Other elements may be indicated according to individual needs, such as subjects with removable prostheses. In addition, supragingival scaling using ultrasonic scaler (Cavitron® Select™ Ultrasonic Scaler, Denstply, New York, USA) and curettes (Millenium, GOLGRAN, São Caetano do Sul, SP, Brazil) are performed. The plaque index is repeated 1 week after OHI and supragingival plaque removal. Subsequently, each volunteer is randomly allocated to one of the following therapeutic groups: (i) c*ontrol*: SRP +two antibiotic placebo pills three times a day (TID) for 14 days + two probiotic placebo lozenges a day for 90 days; (ii) *probiotic* (test 1): SRP + two antibiotic placebo pills TID for 14 days + two probiotic lozenges a day for 90 days; (iii) *antibiotic* (test 2): SRP + MTZ 400 mg and AMX 500 mg TID for 14 days + two probiotic placebo lozenges a day for 90 days; and (iv) *antibiotic + probiotic* (test 3): SRP + MTZ 400 mg and AMX 500 mg TID for 14 days + two probiotic lozenges a day for 90 days (Figs. 1 and 2).

The administration of all medications (probiotic and antibiotics) and placebos starts immediately after the first SRP session. Antibiotics (or placebos) are given TID for 14 days and include two tablets, one of MTZ and one of AMX. Antibiotics and placebos are being specially prepared for this study by the same pharmacy (*Gallen Pharmacy and Manipulation* Ltda, Maringá, PR, Brazil). All tablets have the same color and size and are stored in properly coded opaque plastic bottles, containing 22 capsules/lozenges. Volunteers, therapists, and examiners are blind to the interventions applied.

Probiotic (or placebo) are administered two times per day for 90 days and include one lozenge every 12 h after brushing and flossing. The second lozenge is administered just before going to sleep. The lozenges are slowly dissolved in the volunteer’s oral cavity. Probiotic and placebo are being prepared for this study by *BioGaia AB* (Stockholm, Sweden). Probiotic lozenges contain 2 different strains of *Lactobacillus reuteri*: *L. reuteri* DSM 17938 and *L. reuteri* ATCC (PTA 5289), each at a minimum amount of 1 × 10^8^ CFU per lozenge. Placebo probiotic lozenges are identical to the active lozenges in taste, texture, and shape, but they do not contain *L. reuteri*. All lozenges are kept refrigerated at a temperature below 25 °C until they are handed to the patient.

SRP is performed using Gracey curettes (conventional and mini-fives) 5/6, 7/8, 11/12, and 13/14 under local anesthesia, and the treatment is completed in four to six sessions of approximately 1 h, distributed over a period of 14 days. At the end of each session, the clinical coordinator of each center evaluates the effectiveness of SRP using the outcome “smoothness of the scaled roots.” All volunteers will receive clinical monitoring at baseline and at 3, 6, and 12 months post-therapies. In addition, all subjects will receive periodontal maintenance every 3 months post-treatment until the end of the study. These sessions include OHI and supragingival/subgingival biofilm/calculus removal, as necessary. If any participant shows periodontal attachment loss ≥ 2 mm in 3 or more sites during the maintenance phase, they will be excluded from the study and referred for periodontal re-treatment.

### Monitoring of compliance and adverse events

A study assistant in each clinical center is monitoring the compliance with intake of probiotic/antibiotics/placebos by calling the patients 2 times a week during the first 3 weeks of medication intake. After that, participants are monitored once a week for the next 9 weeks. Volunteers should take 21 antibiotics tablets in the first week and 21 in the second week. They are instructed to return the bottles at beginning of the second week of treatment and receive new bottles containing the same amount of medication to take during the second week. Volunteers are not informed about the total number of tablets in each bottle; therefore, at the end of each week, it is possible to confirm if any residual capsule (22nd) is left in the bottle, to enable compliance to be monitored. Subjects are also asked to return the bottles at 45 days and 90 days post-SRP, and the bottles are checked. On the 14th and at 90 days of the medication intake, subjects answer a questionnaire about any self-perceived side effects.

### Clinical examination (baseline, 3, 6, and 12 months)

Two calibrated examiners, one from each center (UNG and UFPR), are performing the clinical evaluations and collection of biofilm, GCF, and blood samples. The following periodontal parameters are being evaluated: visible plaque [52], gingival bleeding (0/1), BOP (0/1), SUP (0/1), PD (mm), and CAL (mm) at six sites per tooth (mesiobuccal, buccal, distobuccal, distolingual/palatal, lingual/palatal and mesiolingual/palatal) in all teeth, excluding third molars. PD and CAL measurements are rounded to the nearest millimeter using a North Carolina periodontal probe (Hu-Friedy, Chicago, IL, USA).

#### Investigator’ calibration

The two examiners (B.R.V. and G.M.S.S.) were trained and calibrated prior to the trial, in order to achieve maximum reproducibility in the measurements. The calibration exercise for both centers was conducted at UNG before the beginning of the study, as previously described [53]. This exercise will be repeated once a year. Briefly, the standard error of measurement for continuous periodontal clinical parameters (PD and CAL) is evaluated. For the other clinical variables, the average level of agreement between the examiners is determined and considered satisfactory when the value is higher than 90% (Kappa test).

### Microbiological monitoring (baseline, 3, 6, and 12 months)

#### Sample collection

Nine subgingival samples are being collected per volunteer, three in each of the following categories: shallow (PD ≤ 3 mm), moderate (PD = 4–6 mm), and deep (PD ≥ 7 mm). The selected sites are located on non-contiguous interproximal teeth, preferably distributed across the four quadrants and subset according to baseline PD. Teeth with open margins and overhangs, furcation lesions, extensive caries, and/or endo-periodontal lesions are not selected for sampling. After recording the clinical parameters, supragingival plaque is removed and the subgingival samples are collected with individual sterile mini-Gracey curettes #11–12, positioned at the most apical portion of the subgingival sulcus/pocket and with a single apical-coronal movement. The samples are immediately placed in separate Eppendorf tubes containing 150 ul of TE buffer solution (10 mM Tris-HCl, 1 mM EDTA, pH 7.6), and then 100 ul of 0.5 M NaOH is added to each tube and the samples are dispersed using a vortex mixer. The samples are being stored at − 20 °C. The tubes containing the samples are previously identified with the volunteer’s code, date, and site collected.

#### Processing of microbiological samples

Counts and proportion of 40 bacterial species will be determined in each sample, using the Checkerboard DNA–DNA hybridization technique [54, 55]. The microbiological analysis will be entirely performed at the Laboratory of Microbiology of UNG. Briefly, the suspensions containing the bacterial biofilm samples will be boiled for 10 min and neutralized using 0.8 ml of 5 M ammonium acetate, and the DNA is released in the solution. The released DNA will be then placed into the extended slots of a Minislot 30 apparatus (Immunetics, Cambridge, MA, USA) concentrated on a 15 × 15 cm positively charged nylon membrane (Boehringer Mannheim, Indianapolis, IN, USA) and fixed to the membrane by baking it at 120 °C for 20 min. Subsequently, the membrane will be placed in a Miniblotter 45 (Immunetics, Cambridge, MA, USA) with the lanes of DNA at 90^0^ to the lanes of the device. Digoxigenin-labeled whole genomic DNA probes for 40 bacterial species are hybridized in individual lanes of the Miniblotter 45. After hybridization, the membranes are washed in highly astringent solution and the DNA probes are detected using the antibody to digoxigenin conjugated with alkaline phosphatase and chemiluminescence detection. The last two lanes in each run contain standards at concentrations of 10^5^ and 10^6^ cells of each species. Signals will be evaluated visually by comparison with the standards for the test species on the same membrane by a calibrated examiner (k test = 93%). The sensitivity of this assay will be adjusted to allow detection of 10^4^ cells of a given species by adjusting the concentration of each DNA probe [54–56].

### Immunological monitoring (baseline and 12 months)

#### Serum and GCF sampling

Peripheral blood samples and GCF are being collected 1 week after clinical examination. Two non-contiguous diseased sites (i.e., PD and CAL > 5 mm, BoP and no furcation involvement) and two non-contiguous healthy sites (i.e., PD and CAL < 4 mm without BOP and/or gingival bleeding) are being randomly chosen per patient for GCF sampling (among the same sites selected for microbiological sampling). After removal of the supragingival biofilm, the selected sites are isolated with cotton rolls and gently dried with an air syringe to eliminate saliva. GCF is collected by inserting standard paper strips (Periopaper, Oraflow Inc., Smithtown, NY, USA) up to approximately 2 mm into the sulcus/pocket for 30 s. Strips visually contaminated with blood are discarded. The GCF volume is measured in a calibrated device (Periotron 8000, Proflow Inc., Amityville, NY, USA), and the readings are converted into an actual volume (microliter, μl) by reference to a standard curve. Strips from the four selected sites are immediately placed into separate microcentrifuge tubes and stored at − 80 °C for subsequent assays. Peripheral blood samples are into an appropriate tube (Serum BD Vacutainer Plus Plastic Serum Tubes, BD, Franklin Lakes, NJ). Fasted samples are obtained in the morning for all patients. Immediately after blood collection, the serum is separated from blood by centrifugation (10 min at 1300 rpm) and stored in aliquots at − 80 °C.

### Processing of serum and GCF samples

The tubes containing the samples will be vortexed for 15 s and centrifuged for 5 min at 1500×*g* in order to elute. Samples will be analyzed using a Multiplex Bead Immunoassay (MAGPIX® System, Merck Millipore, Billerica, MA, USA) by means of a multiplex fluorescent bead-based immunoassay for 17 cyto/chemokines (eotaxin, GCSF, GM-CSF, interferon (IFN)-γ, IL-1β, IL-2, IL-6, IL-7, IL-8, IL-10, IL-12, protein chemoattractant monocyte [MCP]-1, MIP-1α, and tumor necrosis factor [TNF]-α) using commercially available kits (Milliplex Human Cytokine/Chemokine, EMD Millipore, Billerica, MA, USA) and a plate reader (Luminex. 100TM, EMD Millipore, Billerica, MA, USA), in accordance with the manufacturer’s recommendations. The amount of protein in each sample will extrapolate from standards using an appropriate software (Beadview EMD Millipore, Billerica, MA, USA). The minimum detectable concentrations for eotaxin, G-CSF, GM-CSF, IFN-γ, IL-1β, IL-2, IL-6, IL-7, IL-8, IL-10, IL-12, MCP-1, MIP-1α, and TNF-α are as follows: 1.2 pg/ml, 0.5 pg/ml, 9.5 pg/ml, 0.1 pg/ml, 0.4 pg/ml, 0.3 pg/ml, 0.3 pg/ml, 1.8 pg/ml, 0.2 pg/ml, 0.3 pg/ml, 10.5 pg/ml, 0.9 pg/ml, 3.5 pg/ml, and 0.1 pg/ml, respectively. The results will be reported as total amount (pg/site/30 s) and concentrations of cyto/chemokines per volume of GCF (pg/μl) and serum (pg/ml).

### Primary and secondary outcome variables

The primary outcome variable is the difference among groups for the percentage of volunteers achieving the clinical endpoint for therapy (≤ 4 sites with PD ≥ 5 mm) at 1 year post-treatment [57].

Secondary outcome variables are as follows: difference between baseline and 12 months post-therapy for mean CAL gain and PD reduction (in the full mouth and in different PD categories), mean number and percentage of volunteers with 0, 1–2, or ≥ 3 sites with different thresholds of residual pockets (e.g., PD ≥ 6 and ≥ 7 mm) at 12 months post-therapy; mean number (and mean reductions) of different thresholds of residual pockets at all post-treatment time points; difference among groups for the percentage of volunteers achieving different thresholds of BOP (e.g., ≤ 10%, 20% and 30% of bleeding sites in the mouth) at 1 year post-treatment; differences in the occurrence of adverse events among therapeutic groups; and differences in the counts and proportions of 40 bacterial species in the biofilm samples at all post-treatment time points and in the levels of 17 chemokines in the GCF and peripheral blood samples at 1 year post-therapy.

### Sample size calculation

The ideal sample size to assure adequate power for this study was based on the percentage of volunteers achieving “≤ 4 sites with PD ≥ 5 mm” at 1 year post-treatment [57]. Considering a difference of 44 percentage points between groups for volunteers achieving this clinical endpoint at 1 year post-treatment [5] and a significance level of 5%, 17 subjects per group would be necessary to provide a power of 80%. Considering an attrition of 25%, it was established that at least 22 subjects should be included in each treatment group. Considering a difference of 30 percentage points between groups for volunteers achieving this clinical endpoint at 1-year post-treatment and a significance level of 5% 37 subjects per group would be necessary to provide a power of 80%. Considering an attrition rate of 15%, it was established that 44 volunteers should be included in each treatment group (total 176 subjects).

### Randomization

The study coordinator (M.Fe.), who is not involved in the inclusion and treatment of patients, assigned the study participants to one of the four treatment groups by means of a computer-generated random sequence (Random Allocation Software, http://random-allocation-software.soft). Randomization was stratified by center using permuted blocks of 4, 8, and 12. The study coordinator also organizes the bottles with the antibiotics tablets in opaque plastic bags labeled with the volunteer’s number. In addition, one clinical investigator and the study coordinator sent the randomization list to the *BioGaia AB Company* (Stockholm, Sweden). The company made and packed the probiotic (and placebo) lozenges in boxes labeled with each volunteer’s number. The bags are handed directly to the volunteer by the study coordinator. This sequence of procedures assures allocation concealment.

### Data collection and management

Clinical data are entered directly onto electronic spreadsheets (Microsoft® Excel 2011®, version 14.4.8, Copyright© 1990, Microsoft, Santa Rosa, California, USA), at the time of clinical examination, by a single investigator in each center. Data quality will be validated by checking missing data, out-of-range values, and invalid responses. The analyses will be conducted using a statistical program developed by Sigmund Socransky (Forsyth, USA), as well as SAS and GraphPad Prism (version 7.0) software.

### Statistical analysis

#### Clinical monitoring

The chi-square test will be used to compare the differences in the frequency of gender and to compare the differences in the frequency of subjects achieving the clinical endpoint for treatment (≤ 4 sites with PD ≥ 5 mm) at 1-year (primary outcome variable) and of self-perceived adverse effects. Each individual continue/categorical variable will be computed per subject and then across subjects in each group. The significance of clinical differences over the course of the study will be sought using repeated measures ANOVA or Friedman multiple comparison tests (and their respective post-hoc tests, when necessary), and at each time point (among groups), using either ANOVA or Kruskal-Wallis multiple comparison tests (and their respective post-hoc tests when necessary) or ANCOVA with adjustments for the baseline values. Parametric or non-parametric tests will be chosen based on the normality of the data. A stepwise forward logistic regression analysis will be performed to investigate the impact of predictor variables on the clinical endpoint for treatment, i.e., “presence of ≤ 4 sites with PD ≥ 5 mm at 1-year post-therapy (yes/no).” The Number Needed to Treat (NNT) with each treatment protocol in order to obtain treatment success (≤ 4 sites with PD ≥ 5 mm) will be calculated using the following formula: NNT = 1/ARR, where ARR = |CER − EER| and CER = control group event rate and EER = experimental group event rate (http://ktclearinghouse.ca/cebm/glossary/nnt). The level of significance will be set at 5%. The data will be evaluated using intention-to-treat analysis with last observation carried forward.

#### Microbiological and immunological monitoring

Microbiological data will be expressed in counts (levels) and proportion counts of DNA probes. Mean counts will be expressed as counts × 10^5^. Microbial data will be calculated at each site for each volunteer and then across volunteers within each group, at each time point. The sum of the mean proportions of individual bacterial species will be computed for each microbial complex described by Socransky et al. [58]. The concentration and total amount of each cytokine/chemokine in the GCF will be assessed per volunteer and then per volunteer within each group at each study time point. Immunological data will be calculated at each site for each volunteer and then across volunteers within each group, at each time point. The significance of differences in microbiological and immunological parameters over the course of the study will be sought using repeated measures ANOVA or Friedman multiple comparison tests (and their respective post-hoc tests, when necessary) and at each time point (among groups) using either ANOVA or Kruskal-Wallis multiple comparison tests (and their respective post-hoc tests when necessary) or ANCOVA with adjustments for the baseline values. Parametric or non-parametric tests will be chosen based on the normality of the data.

## Trial status

The study protocol was approved by Guarulhos University (CAAE: 74049717.7.1001.5506) and Federal University of Parana (CAAE: 74049717.7.3001.0102) Ethics Committees on October 3 (2017) and February 28 (2018) respectively.

The recruitment of participants started on November 5 (2018), and the estimated study completion date is December 30 (2021).

## Supplementary Information

**Additional file 1.** SPIRIT 2013 Checklist: Recommended items to address in a clinical trial protocol and related documents*.

**Additional file 2.** Informed consent term.

## Data Availability

The authors of this manuscript declare that the study protocol was registered at *ClinicalTrials.gov* with register number NCT03733379 on November 7, 2018. The data during the current study will be available in the following *clinical trial* website: https://clinicaltrials.gov/ct2/show/NCT03733379?term=Retamal-Valdes&draw=2&rank=1

## Abbreviations

SRP: Scaling and root planing
BOP: Bleeding on probing
SUP: Suppuration
PD: Probing depth
MTZ: Metronidazole
AMX: Amoxicillin
RCT: Randomized clinical trial
UNG: Guarulhos University
UFPR: Federal University of Parana
SPIRIT: Standard Protocol Items: Recommendations for Interventional Studies
CAL: Clinical attachment level
GCF: Gingival crevicular fluid
OHI: Oral hygiene instruction
TID: Thrice a day
ATCC: American Type Culture Collection
CFU: Colony forming unit
M: Molar concentration
DNA: Deoxyribonucleic acid
GEE: Generalized estimating equation
