# Supplementary material for: Clinical, microbiological, and immunological effects of systemic probiotics in periodontal treatment: study protocol for a randomized controlled trial
Source: Trials. 2021 Apr 15;22:283. doi: 10.1186/s13063-021-05246-0 (PMC8048221; doi:10.1186/s13063-021-05246-0)
Supplement: Supplementary file 2 — Additional file 2. Informed consent term. [file 13063_2021_5246_MOESM2_ESM.pdf]

UNG UNIVERSIDADE | ser educacional

Anexo I:

TERMO DE CONSENTIMENTO LIVRE E ESCLARECIDO

TÍTULO DO ESTUDO: "EFEITOS CLÍNICOS, MICROBIOLÓGICOS E IMUNOLÓGICOS DO USO ADJUNTO DE PROBIÓTICOS SISTÊMICOS NO TRATAMENTO PERIODONTAL"

INVESTIGADOR PRINCIPAL: Profa. Dra. Magda Feres
INSTITUIÇÃO: Centro de Pós-Graduação e Pesquisa. Universidade Guarulhos (UNG).
ENDEREÇO: Praça Tereza Cristina 229 - Centro - Guarulhos - Estado de São Paulo. CEP: 07023-070
TELEFONE: (11) 24641684
INSTITUIÇÃO CO-PARTICIPANTE: Universidade Federal de Paraná (UFPR)
OUTROS INVESTIGADORES: Prof[a]. Dr[a]. Luciene Figueiredo (UNG), Prof. Dr. Marcelo Faveri (UNG), Belén Retamal-Valdes (UNG), Prof[a]. Dr[a]. Geisla Mary Soares (UFPR), Natalie Temporão (UFPR).

Iniciais do participante: |_|_|_|_|

Data de nascimento do participante: |_|_| |_|_| |_|_|_|_| dd/mm/aaaa

Número do participante: |_|_|_|_|

PARTICIPAÇÃO VOLUNTÁRIA

Por esse instrumento particular declaro, para os devidos fins éticos e legais, que concordo em absoluta consciência com os procedimentos a que vou me submeter para realização do presente projeto de pesquisa, consistindo de um estudo clínico a longo prazo. Estou ciente que minha participação no estudo é voluntária e que posso retirar meu consentimento em qualquer momento da pesquisa, sem prejuízos à continuidade do tratamento.

OBJETIVOS DO ESTUDO

Fui esclarecido que o objetivo e principal justificativa dessa pesquisa será avaliar os efeitos de um probiótico (espécie bacteriana *Lactobacillus reuteri*) no tratamento da periodontite, doença infecciosa que atinge a gengiva e o osso ao redor dos dentes devido principalmente ao acúmulo de bactérias sobre os dentes. Probiótico é uma terapia natural feito com lactobacillus vivos, a mesma composição de iogurtes disponíveis no mercado.

PARTICIPAÇÃO NO ESTUDO

Estou ciente que o estudo será conduzido em dois centros de pesquisa brasileiros, na Universidade Guarulhos (UNG) e na Universidade Federal de Paraná (UFPR), nos quais serão selecionados 176 participantes com periodontite crônica. Fui informado que o centro onde eu receber a avaliação inicial será o mesmo no qual eu receberei tratamento e posterior acompanhamento. Fui esclarecido que para participar desse estudo, não posso ser fumante e/ou diabético, estar tomando antibiótico e/ou suplementos probióticos, anti-inflamatórios de forma crônica, ou anti-sépticos orais nos últimos 6 meses, não posso ter histórico de tratamento periodontal prévio nos últimos 12 meses, e não posso ter alergia ao metronidazol e/ou à penicilina ou aos probióticos; e quando mulher, não posso estar grávida ou amamentando.

PROCEDIMENTOS

Fui informado que serei submetido aos exames completos dos dentes e da gengiva durante um período de um ano, antes e após o tratamento da periodontite, e que os atendimentos serão efetuados por profissionais formados em odontologia. Estou ciente de que nestas consultas, será feita uma avaliação clínica periodontal completa, e uma pequena quantidade de biofilme (acúmulo de bactérias sobre os dentes), fluido gengival (líquido que sai do espaço entre a gengiva e o dente) e sangue serão coletados para realização dos exames microbiológico (identificação e quantificação bacteriana) e imunológico (avaliação da inflamação). Todas as amostras coletadas serão armazenadas sob refrigeração no Laboratório de Pesquisa II de Odontologia da UnG para posterior análise.

Fui esclarecido que o tratamento da periodontite incluirá a raspagem das superfícies dos dentes (coroa e raiz), o uso de antibióticos (metronidazol 3x/dia e amoxicilina 3x/dia) durante 14 dias e/ou probióticos (espécie bacteriana *Lactobacillus reuteri*) para a eliminação das bactérias presentes na placa dentária causadora da periodontite. Estou ciente que alguns participantes receberão dois antibióticos e/ou probióticos e outros receberão os chamados "medicamentos placebos" – sem a substância química ativa, e que essa escolha será realizada ao acaso, e durante a realização do estudo e eu não poderei ser informado sobre qual medicamento recebi.

BENEFÍCIOS

Fui esclarecido que a principal terapia empregada será o procedimento de raspagem e alisamento radicular (forma mais comum de tratamento da infeção periodontal, não existindo tratamento alternativo), que será o principal benefício direto que eu receberei do estudo. Além disso, fui esclarecido que receberei informações sobre como realizar higiene oral e sua importância para a saúde bucal e geral.

RISCOS E DESCONFORTOS

Estou ciente que a participação neste estudo envolve um risco leve. Serei submetido a coleta de biofilme e fluido gengival que não são invasivas e de sangue que é uma técnica invasiva. Entretanto, fui comunicado que embora a coleta de sangue pode gerar desconforto, ansiedade e algumas complicações, a mesma será realizada por um profissional competente, treinado e apto a manejar as possíveis complicações relacionadas com ela. Fui informado que todos os participantes do estudo, tanto os que receberão os antibióticos, probióticos ou placebo serão questionados, pelo 2 vezes por semana no período de medicação e durante as primeiras três semanas, por contato telefônico, sobre a existência de qualquer efeito colateral como enjôo, dor de cabeça, gosto ruim, diarreia, desconforto abdominal ou reação alérgica/hipersensibilidade. Depois disso, estou ciente que um assistente da pesquisa irá monitorar o consumo dos probiótico/antibiótico/placebo, por contato telefônico, uma vez por semana durante as próximas 9 semanas. Estou ciente que deverei retornar ao centro de pesquisa aos 14, 45 e 90 dias após a realização de raspagem e alisamento radicular para entrega dos frascos contendo os produtos e que aos 14 e 90 dias da medicação, responderei um questionário sobre possíveis efeitos adversos das medicações. Estou ciente que no caso da ocorrência de efeitos indesejáveis, ou mesmo de outros sinais/sintomas incomuns, eu preciso relatar ao pesquisador responsável, e, se for necessário, os medicamentos serão suspensos imediatamente e serei imediatamente afastado(a) do estudo, sem comprometimento ao tratamento gratuito da periodontite ou encaminhamentos para outras especialidades.

TEMPO DOS PROCEDIMENTOS

Foi informado que o tratamento planejado será realizado entre 4 a 6 sessões de 1 hora e finalizada em 14 dias no máximo. Fui esclarecido que o estudo terá uma duração de aproximadamente 3 anos e estou ciente que eu devo retornar aproximadamente 4 vezes ao centro de pesquisa após o tratamento para sessões de manutenção periodontal.

DESPESAS E PAGAMENTOS

Estou ciente que eu não terei custos pelo tratamento recebido, bem como os medicamentos ou os exames de avaliação durante o estudo e, não receberei nenhuma remuneração por participar dele.

GARANTIA DO SIGILO

Fui informado que tenho a garantia do sigilo que assegura minha privacidade no estudo, e uma vez que os dados sejam obtidos, estes poderão ser divulgados em revistas e congressos científicos, mas sem a minha identidade como participante da pesquisa.

DIREITO DE DESISTIR

Estou ciente que terei a garantia de receber novos esclarecimentos que julgar necessários antes e durante o curso da pesquisa, e também terei a plena liberdade para recusar a participação na referida pesquisa a qualquer momento, sem prejuízo algum ou comprometimento do tratamento.

REUTILIZAÇÃO DOS DADOS OU MATERIAL BIOLÓGICO

Eu fui consultado e autorizo a utilização de dados ou material biológico (biofilme, fluido gengival e sangue) em outra pesquisa, sem ter que ser novamente consultado desde que a nova pesquisa seja aprovada pelo Comitê de Ética em Pesquisa da instituição.

PARA MULHERES EM IDADE FÉRTIL

Além disso, caso eu engravide o uso dos antibióticos também será interrompido. Estou ciente que nestes casos, eu também serei imediatamente afastada do estudo, sem comprometimento ao tratamento gratuito da periodontite ou encaminhamentos para outras especialidades.

INDENIZAÇÃO DIANTE DE EVENTUAIS DANOS DECORRENTES DA PESQUISA

Foi informado que a participação no estudo não causará despesas ao mesmo, não havendo, deste modo, previsão de ressarcimento. Com relação às formas de indenização e às medidas de reparo de dano eventual, estou ciente que a participação neste estudo envolve um risco leve, pelo qual não há previsão de formas de indenização. Mas fui informado que eventuais danos resultantes da participação na pesquisa são passíveis de reparação pelos pesquisadores, ainda que não sejam previstos.

CONFIDENCIALIDADE DOS DADOS

Estou ciente que para a tabulação dos dados cada participante do estudo receberá um código, e as amostras coletadas também serão codificadas antes do processamento laboratorial de forma que apenas os pesquisadores envolvidos na coleta de dados terão o conhecimento da identificação dos participantes do estudo.

INFORMAÇÕES DE CONTATO

Foi informado que o resultado do tratamento será dirigido pela Prof^{a}. Dr^{a}. Magda Feres (Telefone: (11) 2464-1726) da UNG, à qual terei acesso para esclarecimentos de eventuais dúvidas ao longo do estudo. Estou ciente que todas as normas de ética do presente estudo estão de acordo com as diretrizes e normas do Conselho Nacional de Saúde na Resolução n° 466/12.

Mas, se eu tiver alguma consideração ou dúvida, eu também posso entrar em contato com o Comitê de Ética em Pesquisa (CEP) da UNG no Prédio do Centro de Pós-Graduação e Pesquisa (CEPPE), Praça Tereza Cristina, n.º 229 - Centro – Guarulhos, comite.etica@ung.br e com a Secretaria do Comitê de Ética da UNG.

CONSENTIMENTO DO PACIENTE

Eu confirmo que discuti até estar satisfeito(a) sobre a minha decisão em participar nesse estudo: **"EFEITOS CLÍNICOS, MICROBIOLÓGICOS E IMUNOLÓGICOS DO USO ADJUNTO DE PROBIÓTICOS SISTÊMICOS NO TRATAMENTO PERIODONTAL"**. Ficaram claros para mim quais são os propósitos do estudo, os procedimentos a serem realizados, seus desconfortos e riscos, as garantias de confidencialidade e de esclarecimentos permanentes. Ficou claro também que minha participação é isenta de despesas e que tenho garantia do acesso a tratamento ou orientação quando necessário. Concordo voluntariamente em participar deste estudo e poderei retirar o meu consentimento a qualquer momento, antes ou durante o mesmo, sem penalidades ou prejuízo ou perda de qualquer benefício que eu possa ter adquirido, ou no meu atendimento neste serviço. Declaro que recebi uma cópia deste Termo de Consentimento Livre e Esclarecido.

/ /
____
Data

______________________________	______________________________
Nome do participante	Assinatura e RG do participante
______________________________	______________________________
Nome responsável legal (se aplicável)	Assinatura e RG do responsável legal (se aplicável)
______________________________	______________________________
Nome da pessoa que explicou o consentimento	Assinatura da pessoa que explicou o consentimento

____

Prof^a^. Dr^a^. Magda Feres

Pesquisadora Responsável

CRO: 77854

RG: 37.957.403-2

Endereço Profissional:
Universidade de Guarulhos
Centro de Pós-graduação e Pesquisa.
Praça Tereza Cristina 229 - Centro - Guarulhos - Estado de São Paulo
CEP: 07023-070
Telefone: (11) 2464-1726
URL da Homepage: http://www.ung.br
